# Staphylococcal Enterotoxins Dose-Dependently Modulate the Generation of Myeloid-Derived Suppressor Cells

**DOI:** 10.3389/fcimb.2018.00321

**Published:** 2018-09-13

**Authors:** Hartmut Stoll, Michael Ost, Anurag Singh, Roman Mehling, Davide Neri, Iris Schäfer, Ana Velic, Boris Macek, Dorothee Kretschmer, Christopher Weidenmaier, Andreas Hector, Rupert Handgretinger, Friedrich Götz, Andreas Peschel, Dominik Hartl, Nikolaus Rieber

**Affiliations:** ^1^Department of Pediatrics I, University of Tuebingen, Tuebingen, Germany; ^2^Proteome Center Tuebingen, Interfaculty Institute for Cell Biology, University of Tuebingen, Tuebingen, Germany; ^3^Interfaculty Institute of Microbiology and Infection Medicine, University of Tuebingen, Tuebingen, Germany; ^4^German Centre for Infection Research (DZIF), Partner Site Tuebingen, Tuebingen, Germany; ^5^Department of Pediatrics, Kinderklinik Muenchen Schwabing, Klinikum Schwabing, StKM GmbH und Klinikum rechts der Isar, Technical University of Munich, Munich, Germany

**Keywords:** *Staphylococcus aureus*, *S. aureus*, myeloid-derived suppressor cells, MDSC, enterotoxin, granulocytes, T cells, immunomodulation

## Abstract

*Staphylococcus aureus* is one of the major human bacterial pathogens causing a broad spectrum of serious infections. Myeloid-derived suppressor cells (MDSC) represent an innate immune cell subset capable of regulating host-pathogen interactions, yet their role in the pathogenesis of *S. aureus* infections remains incompletely defined. The aim of this study was to determine the influence of different *S. aureus* strains and associated virulence factors on human MDSC generation. Using an *in vitro* MDSC generation assay we demonstrate that low concentrations of supernatants of different *S. aureus* strains led to an induction of functional MDSC, whereas increased concentrations, conversely, reduced MDSC numbers. The concentration-dependent reduction of MDSC correlated with T cell proliferation and cytotoxicity. Several findings supported a role for staphylococcal enterotoxins in modulating MDSC generation. Staphylococcal enterotoxins recapitulated concentration-dependent MDSC induction and inhibition, T cell proliferation and cytotoxicity, while an enterotoxin-deficient *S. aureus* strain largely failed to alter MDSC. Taken together, we identified staphylococcal enterotoxins as main modulators of MDSC generation. The inhibition of MDSC generation by staphylococcal enterotoxins might represent a novel therapeutic target in *S. aureus* infections and beyond in non-infectious conditions, such as cancer.

## Introduction

*Staphylococcus aureus* is one of the major human pathogenic bacteria that can cause a broad spectrum of moderate to severe infections ranging from skin and orthopedic infections to fatal necrotizing pneumonia and sepsis. It is regarded as one of the most frequent causes of morbidity and mortality throughout the world (Lowy, 1998). It frequently causes hyperinflammatory reactions of the host immune system contributing to its high mortality rate in systemic infections. Staphylococci possess a thick peptidoglycan layer, which teichoic acids and polysaccharides are bound to. Teichoic acids at the cell wall include wall teichoic acids (WTA) and lipoteichoic acids (LTA). These act as pathogenicity factors and are established TLR-2 ligands (Travassos et al., 2004). Besides others, staphylococcal toxins comprise enterotoxins and the recently identified phenol-soluble modulins (PSM). Of all the 20 or more Staphylococcal enterotoxins, staphylococcal enterotoxin A and B (SEA and SEB) have been best characterized. They are regarded as super-antigens because of their ability to cross-link MHC class II molecules with T-cell receptors and thereby stimulate large populations of T cells independent of specific antigen binding. This results in massive polyclonal T-cell proliferation and inflammatory cytokine secretion (Pinchuk et al., 2010). PSMs are soluble in phenol and considered important virulence factors. Some of these peptides are capable of lysing human neutrophils (Wang et al., 2007). Especially, highly virulent community-associated methicillin-resistant *S. aureus* (CA-MRSA) strains release large amounts of distinct cytolytic PSM peptides (Peschel and Otto, 2013). Interestingly, PSMs have also been reported as immunomodulatory peptides for dendritic cells leading to reduced T-cell inflammation (Schreiner et al., 2013).

Myeloid-derived suppressor cells (MDSC) represent a novel anti-inflammatory mechanism first described in cancer patients (Schmielau and Finn, 2001). In recent years it has become clear that MDSC also play a critical role in the regulation of different types of inflammation that are not directly associated with cancer, e.g., in infectious diseases (Marigo et al., 2008; Gabrilovich and Nagaraj, 2009). These myeloid cells are characterized by their capacity to potently suppress T-cell responses (Gabrilovich and Nagaraj, 2009). MDSC include two major subsets based on their phenotypical and morphological features: polymorphonuclear (PMN-) and monocytic (M-)MDSC. These subsets show unique, yet partially overlapping functional and biochemical characteristics (Gabrilovich and Nagaraj, 2009; Dumitru et al., 2012; Bronte et al., 2016). Phenotypically, human PMN-MDSC have most consistently been determined as CD33^+^CD11b^+^CD14^−^CD15^+^ and M-MDSC as CD33^+^CD14^+^HLA-DR^low^ (Bronte et al., 2016).

MDSC in the context of host-pathogen interaction have been recently reported for several bacterial pathogens (Ost et al., 2016), e.g., for *Klebsiella pneumoniae* (Poe et al., 2013), *Mycobacterium tuberculosis* (du Plessis et al., 2013), and *Pseudomonas aeruginosa* (Rieber et al., 2013). Previous studies have also provided evidence for a contribution of *S. aureus* on MDSC generation and function: (i) Two research groups reported that MDSC are involved in orthopedic biofilm infections (Heim et al., 2014; Peng et al., 2017). Due to their anti-inflammatory action MDSC contributed to the chronicity of *S. aureus* biofilm infections (Heim et al., 2014). (ii) Tebartz et al. described a predominant immunosuppressive effect of MDSC compared to regulatory T cells for the chronicity of *S. aureus* infections (Tebartz et al., 2015). (iii) On the other hand ameliorated disease courses have also been described under the influence of MDSC, e.g., in mouse models of acute staphylococcal toxic shock syndrome caused by staphylococcal enterotoxin B (Szabo et al., 2016) and of atopic dermatitis with *S. aureus* colonized skin (Skabytska et al., 2014).

Based on these previous findings, we aimed to further determine the impact of different *S. aureus* strains and associated virulence factors on human MDSC generation in this *in vitro* study. Here we demonstrate for the first time that staphylococcal enterotoxins dose-dependently modulate the generation of MDSC. The interaction of staphylococcal enterotoxins with myeloid-derived suppressor cells might play an important role in the overshooting inflammatory reaction frequently seen in systemic *S. aureus* infections.

## Materials and methods

### Bacterial strains, culture conditions, and preparation of staphylococcal supernatants for stimulation assays

In order to analyze *S. aureus*-mediated induction or inhibition of MDSC formation, we used a variety of staphylococcal strains (Table 1). Bacteria were stored as glycerol stocks at −80°C and grown overnight on TSB agar plates at 37°C (casein peptone 17 g/l, soya peptone 3 g/l, glucose 2.5 g/l, dipotassium hydrogen phosphate 2.5 g/l, sodium chloride 5 g/l, Sigma-Aldrich). Single colonies from each strain were inoculated and shaken for 16 h at 130 rpm at 37°C in RPMI 1640 medium (Biochrom) supplemented with 4 mM L-glutamine (Gibco/Life Technologies). Bacterial cells were removed by centrifugation for 30 min at 5,000x g at 4°C and the supernatants were sterile-filtered twice using 0.2 μm non-pyrogenic filters. Equivalent growth of the bacteria was verified by optical density measurements at 600 nm and by CFU counting on TSB agar plates. *P. aeruginosa* was grown overnight in TSB medium instead of RPMI 1640, and supernatants were prepared as described (Rieber et al., 2013). The filtered supernatants were stored in aliquots at −20°C and were used for stimulation experiments.

**Table 1:** Bacterial strains used in this study.

**Strain (substrain)**	**Strain ID *[a]***	**Genotype and description *[b]***	**References**
***S. aureus***			
USA300 (FPR3757)	ATCC BAA-1516, NCBI 451515, NRS482	Referred to as ‘USA300’ in this study; CA-MRSA; *agr+*	McDougal et al., 2003; Diep et al., 2006
USA300 (LAC) Δ*agr*	n.a.	CA-MRSA; deleted *agr* locus	Cheung et al., 2011
USA300 (JE2)	JE2	CA-MRSA; derived from USA300 (LAC) by curing of three plasmids; parental strain of transposon insertion mutants collected in the Nebraska Transposon Mutant Library	Fey et al., 2013
USA300 (JE2) *secA-*	NE66	Tn insertion in preprotein translocase gene SAUSA300_2584	Fey et al., 2013
USA300 (JE2) putative enterotoxin type A-	NE309	Tn insertion in putative enterotoxin type A gene SAUSA300_1559	Fey et al., 2013
USA300 (JE2) *sek-*	NE1255	Tn insertion in enterotoxin K gene SAUSA300_0800	Fey et al., 2013
USA300 (JE2) *seq-*	NE1605	Tn insertion in enterotoxin Q gene SAUSA300_0801	Fey et al., 2013
USA300 (JE2) *selX-*	NE1809	Tn insertion in putative enterotoxin selX gene SAUSA300_0370	Fey et al., 2013
USA300 (SF8300)	n.a.	CA-MRSA	Diep et al., 2008b
USA300 (SF8300) Δ*ACME*	n.a.	Deleted *ACME* locus	Diep et al., 2008b
USA400 (MW2)	ATCC BAA-1707, NCBI 196620, NRS123	CA-MRSA	Baba et al., 2002
N315	NCBI 158879, NRS70	HA-MRSA	Kuroda et al., 2001
Mu50	ATCC 700699, NCBI 158878, NRS1	Vancomycin-intermediate HA-MRSA	Kuroda et al., 2001
COL	NCBI 93062, NRS100	HA-MRSA, *agr* low, *sigB+*	Gill et al., 2005
Newman	ATCC 13420, NCTC 8178, NCBI 426430	MSSA; *agr+; saeRS* constitutively expressed; PVL- phenotype; *fnbA-* and *fnbB-*	Baba et al., 2008
Newman φSa2MW	n.a.	Newman lysogenized with φSa2MW carrying *lukF/S-PV*	Wirtz et al., 2009
PS187	ATCC 15564, NCTC 9754, NCBI 1323662	MSSA; prototype of ST395 lineage; unique WTA structure with a GroP-GalNAc backbone	Winstel et al., 2013
NCTC 8325; RN1	NCTC 8325, NCBI 93061, NRS77	MSSA; parental strain of 8325-4, RN4220, HG003, SA113, RN6390; φ11+ φ12+ φ13+, *agr+, rsbU-, tcaR-*	Novick, 1967
HG003	n.a.	RN1 derivative; φ11+ φ12+ φ13+, *agr+, rsbU* and *tcaR* repaired	Herbert et al., 2010
SA113	ATCC 35556, DSM 4910	RN1 derivative; MNNG mutagenesis; φ11+ φ12+ φ13+, *agr- rsbU- tcaR-*, r- m-	Iordanescu and Surdeanu, 1976
8325-4; RN0450	NRS135	RN1 derivative; UV mutagenesis; φ11- φ12- φ13-, *agr+, rsbU-, tcaR-*	Novick, 1967
RN6390	n.a.	RN1 derivative; UV mutagenesis, φ6390 lysogenized and Tn554 *erm* insertion; φ11- φ12- φ13-, *agr+ rsbU- tcaR-*	Peng et al., 1988
RN4220	NCBI 561307, NRS144, DSM 26309	RN1 derivative; UV and MNNG mutagenesis; φ11- φ12- φ13-, *agr-, rsbU-, tcaR-*, r- m-	Kreiswirth et al., 1983
***S. carnosus***			
TM300	NCBI 396513	Meat starter culture bacterium, apathogenic	Rosenstein et al., 2009
***P. aeruginosa***			
PAO-1	ATCC 15692, NCBI 208964, DSM 22644	MDSC-inducing opportunistic pathogen, persists in cystic fibrosis	Stover et al., 2000

*Strain IDs were taken from American Type Culture Collection (ATCC), National Collection of Type Cultures (NCTC) and from DSMZ, German Collection of Microorganisms and Cell Cultures. JE, NE and NRS numbers correspond to IDs from the Network on Antimicrobial Resistance in Staphylococcus aureus (NARSA) program. Described are relevant genotypic features referring to determination or to regulation of virulence within the scope of this study*.

*FPR3757 and LAC are different isolates from the same PFGE-type (USA300-0114) of S. aureus. JE2 derivative strains display an insertion of the mariner-based bursa aurealis transposon in the respective genes. Disrupted genes are marked with a ‘-’; deleted loci are denoted with a ‘Δ’. ACME, arginine catabolic mobile element; agr, accessory gene regulator; CA, community-acquired; fnbA, Fibronectin binding protein A; fnbB, Fibronectin binding protein B; GalNAc, N-acetyl-d-galactosamine; GroP, glycerophosphate; HA, hospital-acquired; hlb, hemolysin beta; MRSA, Methicillin-resistant S. aureus; MSSA, Methicillin-susceptible S. aureus; PFGE, pulsed-field gel electrophoresis; PVL, Panton-Valentine leukocidin; r– m–, restriction-modification mutant; rsbU – regulator of sigma factor SigB; saeRS, S. aureus exoprotein expression regulatory system; secA, Sec system protein translocase subunit A; sek, Staphylococcal enterotoxin k; seq, Staphylococcal enterotoxin q; SigB, RNA polymerase sigma factor B; tcaR, Teicoplanin-resistance associated HTH-type transcriptional regulator; Tn, transposon; selX- Staphylococcal enterotoxin-like X; WTA, wall teichoic acid; φ, Prophage Phi. n.a., not available*.

### Pre-treatment of staphylococcal supernatants and enterotoxins

Where indicated, staphylococcal supernatants from RPMI overnight cultures were diluted 1:3 in RPMI 1640 and pre-treated with 20 μg/ml proteinase K (Promega) at 130 rpm at 37°C for 16 h. Heat-treatment of staphylococcal supernatants or staphylococcal enterotoxins was performed at 80°C for 20 min.

For size exclusion experiments, Vivaspin 15 concentrators with molecular weight cutoffs (MWCO) of 10,000 or 50,000 were used (Sartorius Stedim Biotech). Staphylococcal supernatants were centrifuged through MWCO 50,000 filters for 30 min at 4.000x g and 4°C, resulting in a 300-fold concentration. The flow-through was then centrifuged in a second step through MWCO 10,000 filters using the same conditions. The flow-through and the residual concentrates were adjusted after each step to the initial volume with the medium used for bacterial growth.

### Isolation of human PBMC

Peripheral blood mononuclear cells (PBMC) were prepared from heparinized human blood samples from healthy volunteers by Ficoll density gradient sedimentation (Lymphocyte separation medium, Biochrom). PBMC were washed twice in RPMI 1640 medium (Biochrom), resuspended in RPMI 1640 supplemented with 10% FCS (Biochrom), 2 mM L-glutamine (Sigma-Aldrich) and 1% penicillin/streptomycin (Biochrom), referred to as “complete medium,” and used in stimulation assays. A viability of >90% was determined for all PBMC preparations by using trypan blue staining.

### MDSC generation assay and flow cytometric analyses

Immediately after isolation PBMC were seeded into 24-well flat bottom plates (Corning) at 7.5 × 10^5^ cells per well in 1.5 ml complete medium, treated with stimulants as indicated, and cultured in a humidified atmosphere at 37°C and 5% CO_2_. After 4 days, medium and stimulants were refreshed. At day 6 of cultivation, all cells were collected from PBMC cultures and washed and resuspended in D-PBS (Sigma-Aldrich). Adherent cells were harvested using non-protease Detachin Cell Detachment Solution (Genlantis). SEA and SEB were purchased from Sigma-Aldrich. Non-bacterial stimulants were granulocyte-macrophage colony-stimulating factor (GM-CSF, 10 ng/ml; Genzyme), *Aspergillus fumigatus* lysate (10 μg/ml; Miltenyi Biotec), and IL-2 (100 U/ml; MBL).

**Figure 1:**
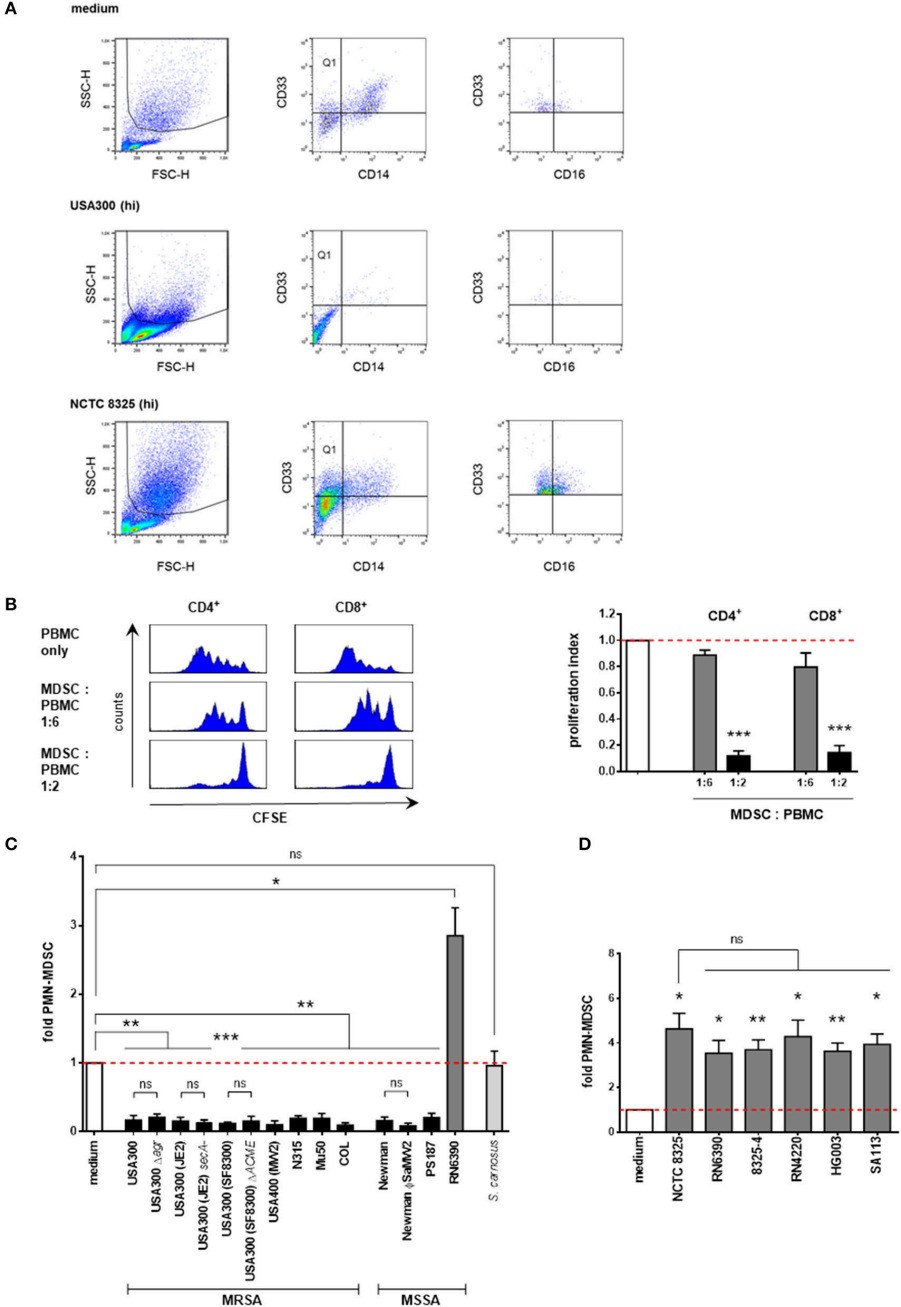
Supernatants from *S. aureus* strains differentially modulate *PMN-like* MDSC levels. PBMC were stimulated with supernatants prepared from overnight cultures of the indicated staphylococcal strains and *PMN-like* MDSC were assessed by flow cytometry. **(A)** Phenotypic determination of *PMN-like* MDSC in PBMC. The granulocytic region was gated in the forward-side-scatter. Afterwards CD33^+^CD14^−^ cells (Quadrant Q1) were gated and *PMN-like* MDSC were determined as SSC^high^CD33^+^CD14^−^ cells. Further flowcytrometric analysis revealed that these granulocytic cells are partly CD16^+^. The dot plots illustrate the modulation of *PMN-like* MDSC mediated by supernatants from NCTC 8325 and USA300 [at 3% (hi) concentration] as compared to medium only. **(B)** *S. aureus*-induced PMN-MDSC dose-dependently suppress T-cell proliferation. *PMN-like* MDSC were induced using 0.02% of USA300 supernatants, isolated by CD33 MACS separation and co-cultured for 4 days with freshly isolated, CFSE-stained PBMC at given ratios. CFSE-fluorescence intensity of CD4^+^ and CD8^+^ T cells was analyzed by flow cytometry. Left panel: Histograms showing suppression of T cell proliferation. Right panel: Bars represent the proliferation index. The values are normalized to the proliferation of CD4^+^ cells or CD8^+^ T cells without addition of MDSC. Bars represent means ± SEM. Differences between MDSC co-cultures and controls were analyzed by a one-sample *t*-test. **(C)** Screening of *S. aureus* supernatants for modulation of *PMN-like* MDSC induction. PBMC were stimulated using 3 vol.% of supernatants prepared from overnight cultures of the indicated staphylococcal strains. Except for RN6390, all tested *S. aureus* strains inhibited the *PMN-like* MDSC formation. *S. aureus* strains are illustrated in black bars except for NCTC 8325 members (shown in dark gray bars). *S. carnosus* is shown in light gray bars. **(D)** Screening of NCTC 8325 derivative strains. All tested members of the NCTC 8325 family consistently induced *PMN-like* MDSC. Bars represent means ± SEM. Differences between stimulations and controls **(C,D)** were analyzed by a one-sample *t*-test. Differences between different wild-type and mutant *S. aureus* strains **(C,D)** were analyzed by a Mann-Whitney test or by an unpaired *t*-test. *p < 0.05; **p < 0.01; ***p < 0.001; ns–not significant.

MDSC numbers in PBMC cultures were quantified by flow cytometric analyses. PMN-MDSC were gated as SSC^high^CD33^+^CD14^−^ cells. These cells were partly positive for CD16 (see Figure 1A for gating strategy). Further representative surface marker staining demonstrated strong positivity for CD11b but negative staining for CD15 (Supplemental Figure 1). Therefore, these granulocytic cells were considered as *PMN-like* MDSC. Total numbers of apoptotic and dead cells were determined by annexin V and propidium iodide staining. For quantification of total live cells, trypan blue staining was performed and living cells were counted in a Neubauer chamber. PE-labeled anti-CD33, APC-labeled anti-CD11b and respective isotype control antibodies were purchased from Miltenyi Biotec; PI, FITC Annexin V, FITC-labeled anti-CD14, APC-labeled anti-CD14, FITC-labeled anti-CD15, PerCP-labeled anti-CD16, APC-labeled anti-CD3, and respective isotype control antibodies were obtained from BD Pharmingen. Flow cytometry was performed using a FACSCalibur (BD), and analyses of cell percentages were performed using BD CellQuest Pro software. The percentage of PMN-MDSC in medium-only cultures was set to 1. Percentages of PMN-MDSC after stimulation are presented as x-fold compared to medium controls. Unstained controls were used to set dot plot quadrants.

### T-cell suppression assay

MDSC were generated from myeloid cells of the PBMC fraction as described above and isolated from cell cultures by magnetic bead cell sorting for CD33 (Miltenyi Biotec). Responder-PBMC were obtained from healthy volunteers' heparinized blood and stained with CFSE (Life Technologies) according to the manufacturer's protocol. CFSE-labeled PBMC were stimulated with 100 U/ml IL-2 (R&D Systems) and 1 μg/ml OKT3 (Janssen-Cilag). Both MDSC and CFSE-labeled PBMC were added to RPMI 1640 medium supplemented with 10% human serum, 2 mM L-glutamine, 100 IU/ml penicillin and 100 mg/ml streptomycin. In a 96-well round bottom plate (Greiner Bio-One), either 10,000/30,000 MDSC or, as a control supplemented medium only, were added to 60,000 PBMC per well. Cells were incubated in a humidified atmosphere at 37°C and 5% CO_2_. On day 4 cells were harvested and stained with anti-CD8a-APC, anti-CD4-PE antibodies (BioLegend), and propidium iodide (BD). PI positive cells were excluded in flow cytometry. CFSE signals of CD4^+^ and CD8^+^ PBMC were analyzed.

### MS analyses of staphylococcal supernatant proteins

#### Preparation of staphylococcal supernatant proteins for MS analyses

To determine their enterotoxin contents, staphylococcal supernatants were prepared the same way as for the stimulation assays above, except that overnight cultures were grown in TSB medium (Sigma-Aldrich) for 14 h to obtain higher yields of supernatant proteins. Equivalent amounts of bacteria were confirmed by optical density measurements at 600 nm and by CFU counting. Proteins were obtained from the supernatants by means of chloroform/methanol precipitation, using 4 vol. methanol, 1 vol. chloroform and 3 vol. H2O_dd_ per vol. overnight culture. After centrifugation for 45 min at 4.800x g and 12°C, the aqueous phase was removed and 6 vol. methanol were added, followed by a second centrifugation step using the same conditions in order to pellet the proteins. The protein pellets were air-dried and stored at −20°C.

#### Tryptic digestion of proteins

For proteome analysis protein pellets were run on a gel, and following a brief Coomassie staining, tryptic digestion of proteins and nano-MS/MS analysis were done as previously described (Burian et al., 2015), except that we used a 230 min segmented gradient.

#### MS data processing and analysis

Acquired MS spectra were processed with MaxQuant software package version 1.2.2.9 (Olsen et al., 2005; Cox and Mann, 2008) with integrated Andromeda search engine. Database search was performed against a target-decoy of all *S. aureus* strains. The database was obtained from UniProt (taxonomy ID 1280), containing 126,225 protein entries and 247 commonly occurring laboratory contaminants. Pursuant to TSB medium contents, we additionally searched against *Bos taurus* (cattle; taxonomy ID 9913) containing 24,240 protein entries, and *Glycine max* (soybean; taxonomy ID 3847) containing 64,601 protein entries, both obtained from UniProt. Endoprotease Trypsin was fixed defined as the protease with a maximum missed cleavage of two. Oxidation of methionines and N-terminal acetylation were specified as variable modifications, whereas carbamidomethylation on cysteines was defined as a fixed modification. Initial maximum allowed mass tolerance was set to 6 ppm (for the survey scan) and 0.5 Da for CID fragment ions. A false discovery rate of 1% was applied at the peptide and protein level. A minimum of two unmodified peptide counts were required for the respective protein quantification. The label-free algorithm was enabled, as was the “match between runs” option (Luber et al., 2010).

### Statistical analysis

Statistical analysis was performed in GraphPad Prism version 6.0 using a one-sample *t*-test, a Mann-Whitney test or an unpaired *t*-test as indicated. In all tests, differences were considered significant at *P* < 0.05 (^*^P < 0.05; ^**^ *P* < 0.01; ^***^ *P* < 0.001; ^****^ *P* < 0.0001).

## Results

### *S. aureus* strains differentially modulate *PMN-like* MDSC

In order to determine the impact of *S. aureus* on the generation of MDSC, we built on an established *in vitro* MDSC generation system (Lechner et al., 2010; Rieber et al., 2013) to induce PMN-MDSC from peripheral blood of healthy donors and to quantify them by flow cytometry. PMN-MDSC were initially gated as cells with high granularity (SSC^high^), bearing the myeloid marker CD33, devoid of the monocytic marker CD14 (representative dot plots Figure 1A) and with the characteristic to suppress T-cell responses (representative data in Figure 1B). Additional flowcytometric analysis revealed that these suppressive myeloid cells are strongly CD11b^+^, partly CD16^+^, and CD15^−^ (Figure 1A and Supplemental Figure S1), why we would rather term them *PMN-like* MDSC.

We initially screened supernatants at concentrations of 3% from a broad range of *S. aureus* strains, including various clinical MRSA and MSSA isolates and several mutant strains affected in their expression of prominent virulence genes for their ability to modulate the generation of MDSC. In addition, we tested supernatants from several established staphylococcal laboratory strains and from another staphylococcal species, *S. carnosus*. All *S. aureus* strains tested abrogated the formation of MDSC, except for strain RN6390, which induced MDSC (Figure 1C). In contrast, the non-pathogenic food-grade bacterium *S. carnosus* did not modulate MDSC cell levels significantly.

Compared to the respective wild-type strain USA300, knock out of the global virulence regulator *Agr*, which regulates a wide variety of virulence determinants (Novick, 2003), did not affect MDSC levels. Also, transposon mutagenesis of *secA*, a component of the Sec pathway for protein secretion (Green and Mecsas, 2016), had no effect in the USA300 substrain JE2.

The arginine catabolic mobile element ACME, a mobile genetic element conferring survival and growth in hosts by providing polyamine resistance (Diep et al., 2008b; Joshi et al., 2011), is predominantly expressed in MRSA strains (Shore et al., 2011). Similarly, the pore-forming pantene-valentine leucocidin (PVL), which displays toxic effects on neutrophils (Genestier et al., 2005) is present in virtually all CA-MRSA strains and in some MSSA strains (Diep et al., 2008a; Wirtz et al., 2009). Both knock out of the ACME locus in USA300 substrain SF8300 and lysogenization of PVL-negative Newman with phage ΦSa2MW, which carries the PVL gene locus lukF/S-PV (Bubeck Wardenburg et al., 2007), had no effect on the generation of MDSC compared to their respective parental strains.

Strain RN6390 is a highly-mutated member of the NCTC 8325 lineage of *S. aureus* strains. Its parental strain NCTC 8325 (also known as RN1) is a well-established laboratory strain commonly used for genetic studies, whereby numerous derivative strains have been engineered by means of mutagenic procedures and by reconstruction of distinct mutagenized gene loci (Herbert et al., 2010; Baek et al., 2013). To test whether any of the genes mutated had an impact on the formation of MDSC, we used supernatants of parental strain NCTC 8325 and its derivative strains RN6390, 8325-4, RN4220, HG003, and SA113 for stimulation (Figure 1D). The parental NCTC 8325 strain has functional agr, sarA, and sae global virulence regulators, however, it is characterized by a deficiency in the acitivity of the important virulence regulator sigma factor SigB, which is due to a deletion in the rsbU gene, and in the regulator gene tcaR, an activator of protein A transcription, both of which have been reconstituted in strain HG003. Moreover, it is deficient in production of beta-hemolysin due to the insertion of prophage ϕ13 in the hlb gene. ϕ13, however, is coding for the IgG, C3b and fibrin degrading staphylokinase. The strains RN6390 and 8325-4 have been cured from ϕ13 and other prophages. RN4220 displays an additional deficiency in production of functional alpha- and delta-hemolysins and a partial inactivation of the global virulence regulator region agr, whereas strain SA113 is another agr mutant derived directly from NCTC 8325. In our *in vitro* MDSC generation system all tested members of the 8325 family induced MDSC to a similar degree (Figure 1D). Taken together, our results suggest that the above mentioned virulence factors are not necessary for modulation of MDSC levels.

The genome of the parental NCTC 8325 strain, but not that of RN6390, has been sequenced and annotated (GenBank accession no. NC_07795.1) and protein databases are available (UniProt Taxon ID 93061). We therefore performed further comparative functional, genomic, and proteomic analyses using NCTC 8325 and the closely phylogenetically related (Baba et al., 2008) and well-characterized clinical isolates USA300, Newman and COL.

### The modulation of *PMN-like* MDSC levels by *S. aureus* supernatants is dose-dependent

**Figure 2:**
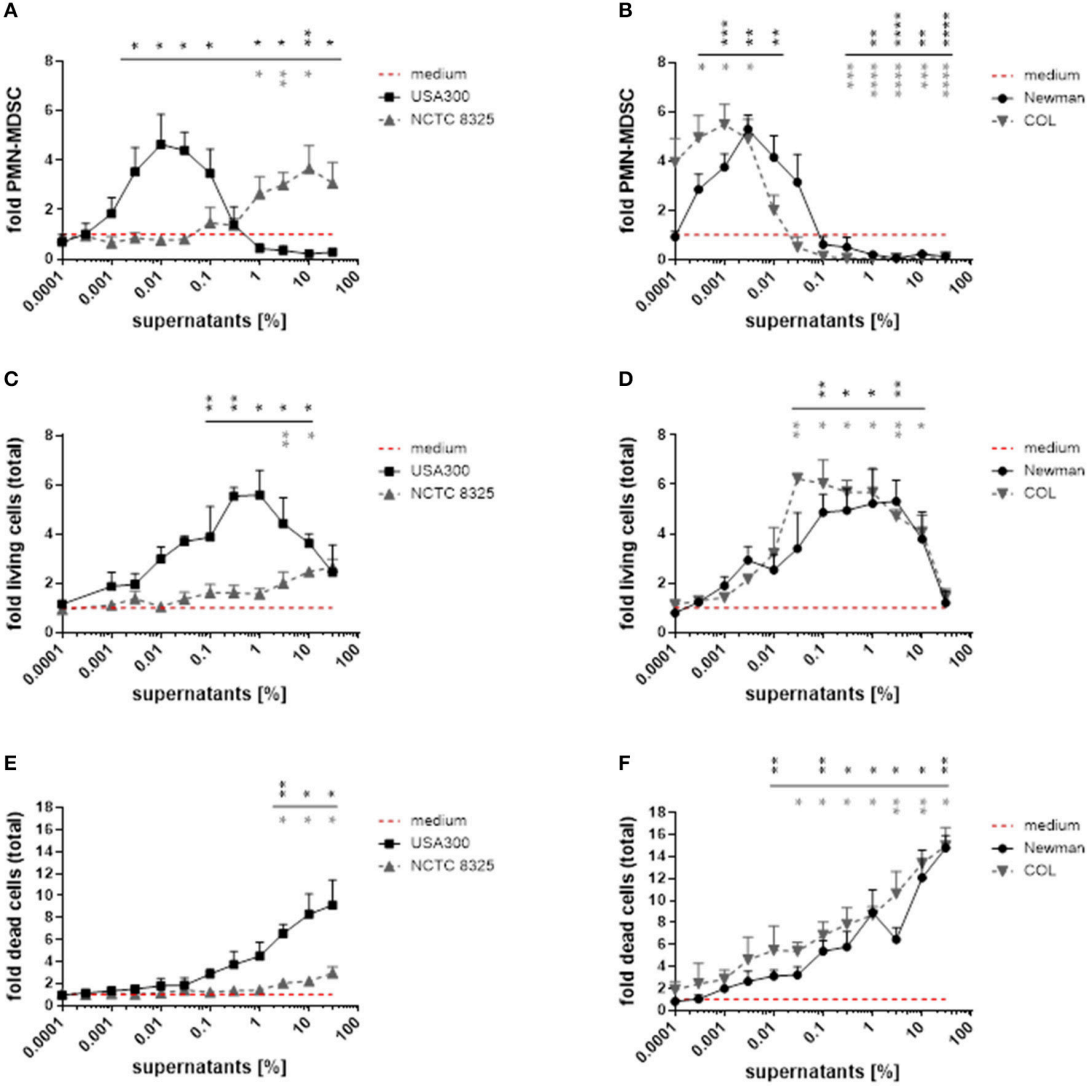
*S. aureus* supernatants dose-dependently modulate *PMN-like* MDSC formation, proliferation and cytotoxic effects in PBMC. PBMC were treated with supernatants from *S. aureus* strains USA300, NCTC 8325, Newman, and COL in a broad concentration range. **(A,B)** *PMN-like* MDSC were quantified by flow cytometry. **(C,D)** Proliferation of total PBMC was assessed by trypan blue staining. **(E,F)** Cell death in total PBMC cultures was measured by PI staining and subsequent flow cytometry. Data are presented as means ± SEM. Differences between stimulations and medium controls were analyzed by a one-sample *t*-test and are depicted in colors referring to the respective curves. *p < 0.05; **p < 0.01; ***p < 0.001; ****p < 0.0001.

By using broad concentration ranges of *S. aureus* supernatants, we demonstrated that both MDSC-inhibiting and MDSC-inducing activities exist within most *S. aureus* strains. Lower concentrations induced PMN-MDSC (peak levels at 0.01 for USA300, 0.003 for Newman, and 0.001% for COL), whereas increased concentrations led to marked suppression of PMN-MDSC when using supernatants from these strains (Figures 2A,B). In contrast, supernatants from strain NCTC 8325 showed virtually no MDSC expanding activity below 0.01% supernatant concentration, peaked PMN-MDSC levels not until 10%, and no suppression was detected even with supernatant concentrations of up to 30%.

### *PMN-like* MDSC modulation correlates both with PBMC proliferation and cytotoxicity

With rising concentrations of the supernatants we observed a concomitant proliferation of the PBMC in culture. Therefore, we did not only determine the absolute numbers of *PMN-like* MDSC, but also quantified total PBMC numbers and discriminated live and dead cells by trypan blue exclusion and PI staining (Figure 2). During a first phase *PMN-like* MDSC numbers raised, while total PBMC numbers increased concurrently and only moderate cell death occurred. During a second phase *PMN-like* MDSC numbers declined, whereas total PBMC still accumulated substantially despite increasing cytotoxicity. During a third phase all myeloid cells were predominantly killed when toxicity exceeded a certain level. With the exception of supernatants from strain NCTC 8325, these dose-dependent phases applied to stimulations with all tested *S. aureus* supernatants. In order to determine if the abrogating effect on MDSC is by inhibiting differentiation into MDSC or by actually killing of MDSC, we performed Annexin/PI staining on gated MDSC. The results in Supplemental Figure 2A point to cytotoxic effects on *PMN-like* MDSC rather than a stop in differentiation into MDSC. Whether this killing effect is direct or indirect via other cell types cannot be clarified within the current study, because most CD33^+^ cells, if isolated, died within three days even in the medium control (Supplemental Figure 2B). The exact mechanism for the ceasing of MDSC remains a subject for future investigations.

### MDSC inhibition outweighs induction by different *S. aureus* supernatants

**Figure 3:**
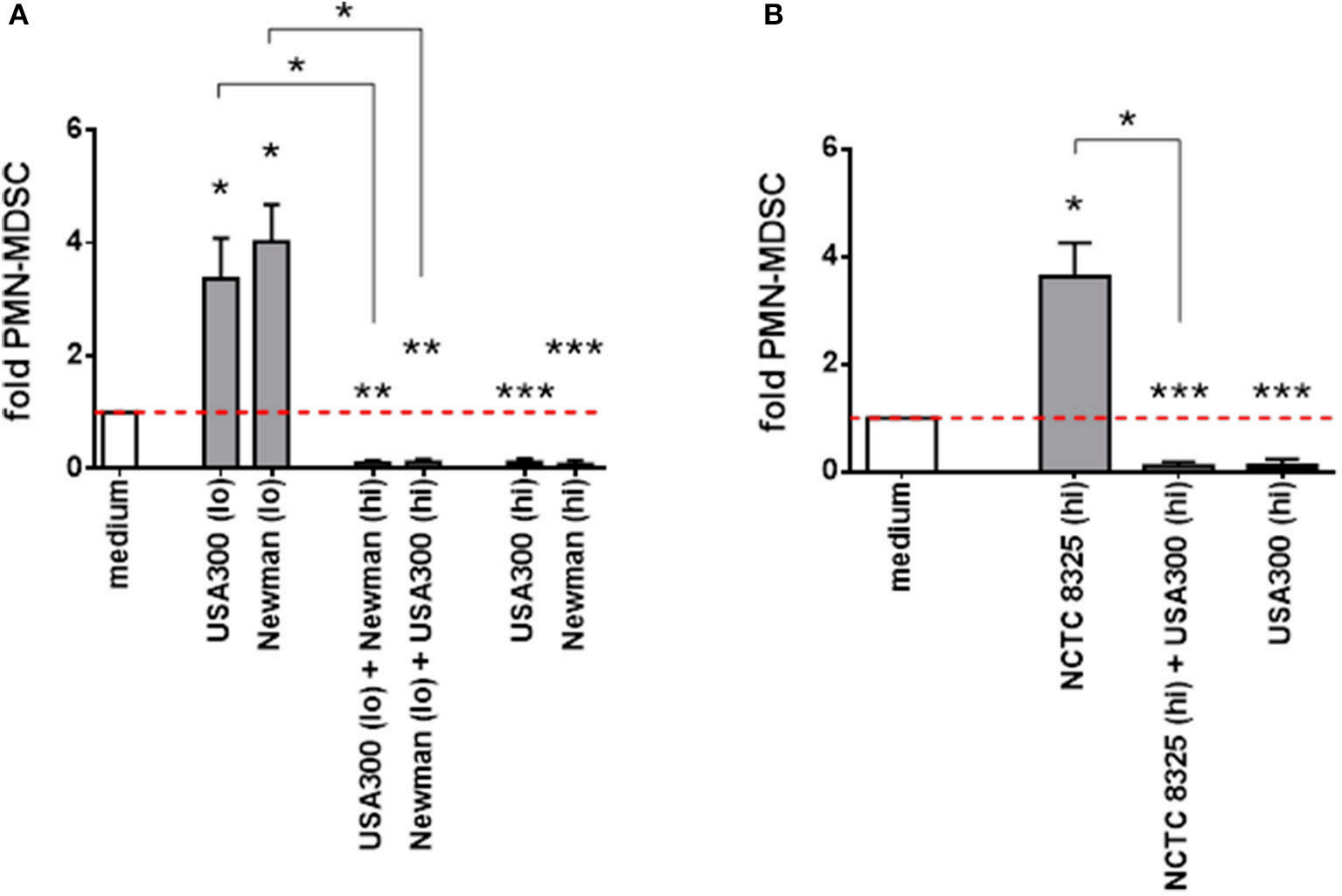
*PMN-like* MDSC inhibition outweighs induction by different *S. aureus* supernatants. PBMC were stimulated simultaneously with high and low doses of supernatants taken from different *S. aureus* strains. *PMN-like* MDSC induction mediated by supernatants from *S. aureus* strains USA300 or Newman **(A)** and from the less active NCTC 8325 strain **(B)** is suppressed by supernatants derived from other strains at concentrations previously shown to be inhibitory. For induction of *PMN-like* MDSC (shown as light gray bars), low concentrations (0.01%) from USA300 or Newman and high concentrations (10%) from NCTC 8325 were used. For suppression (shown as black bars), high concentrations (3%) from USA300 or Newman were used. Mixed stimulations are illustrated as dark gray bars. Data sets are represented as means ± SEM. Differences between stimulations and medium controls were analyzed by a one-sample *t*-test. Differences between single and mixed stimulations were analyzed by a Mann-Whitney test. *p < 0.05; **p < 0.01; ***p < 0.001.

When different *S. aureus* supernatants with divergent effects were mixed, inducing activities were outweighed by suppressive activities (Figure 3A). Strain NCTC 8325 significantly raised *PMN-like* MDSC levels only at high concentrations. This NCTC 8325-mediated MDSC induction was suppressed by inhibitory concentrations of USA300 supernatants (Figure 3B). We concluded that, in terms of modulation of *PMN-like* MDSC, high concentrations of NCTC 8325 supernatants correspond to lower concentrations of supernatants derived from other *S. aureus* strains, and that there might be an universal factor among different *S. aureus* strains for dose-dependent modulation of MDSC.

### *S. aureus* modulates *PMN-like* MDSC through secreted and heat-stable proteins

**Figure 4:**
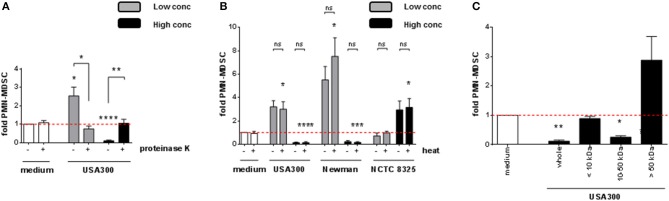
*PMN-like* MDSC levels are modulated by heat-stable *S. aureus* proteins sized 10 to 50 kDa. *S. aureus* supernatants were pre-treated prior to stimulation of PBMC. **(A)** Pre-treatment with 20 μg/ml of proteinase K at 37°C for 16 h, **(B)** heat-treatment at 80°C for 20 min, **(C)** filtration through spin columns with MWCO cut-off pores as indicated. 3% (USA300 or Newman) or 10% (NCTC 8325) of supernatants were used as high concentrations (black bars), 0.01% (all tested strains) were used as low concentrations (gray bars). Data are presented as means ± SEM. Differences between stimulated samples and medium controls were analyzed by a one-sample *t*-test. Differences between different pre-treatment conditions **(A,B)** were analyzed by a Mann-Whitney test. *p < 0.05; **p < 0.01; ***p < 0.001; ****p < 0.0001; ns–not significant.

To better define the nature of the MDSC modulating factor, *S. aureus* supernatants were pre-treated under different conditions before using them for stimulation of PBMC. After digestion with proteinase K, both the MDSC inducing and the MDSC inhibiting activities were abrogated, as shown for USA300 (Figure 4A). Heat-treatment of supernatants did not affect the results (Figure 4B). Size fraction experiments using spin columns with respective MWCO cut off pores revealed that the MDSC modulating activities resided in size fractions ranging from 10 to 50 kDa. Residual stimulating activities were irregularly observed in the fraction of larger molecules, however without statistical significance (Figure 4C).

Our results indicated that the major MDSC modulating factors of *S. aureus* are secreted, heat-stable, proteinaceous molecules with molecular masses between 10 and 50 kDa and are produced by pathogenic rather than by apathogenic staphylococci. Furthermore, for all strains except the NCTC 8325 family, we observed a decline of *PMN-like* MDSC at increasing supernatant concentrations, whereas total PBMC numbers were still growing. By immunophenotyping this proliferation was attributed amongst others to a considerable growth of CD3^+^ T-cells. The observation that heat-stable, proteinaceous factors between 10 and 50 kDa in size are responsible for modulation of *PMN-like* MDSC levels, and that a considerable T-cell growth occurred concomitantly to the decline in *PMN-like* MDSC numbers, led us to investigate if superantigenic staphylococcal enterotoxins may be the key factor involved in *S. aureus*-mediated modulation of MDSC.

### *S. aureus* enterotoxins dose-dependently modulate *PMN-like* MDSC

**Figure 5:**
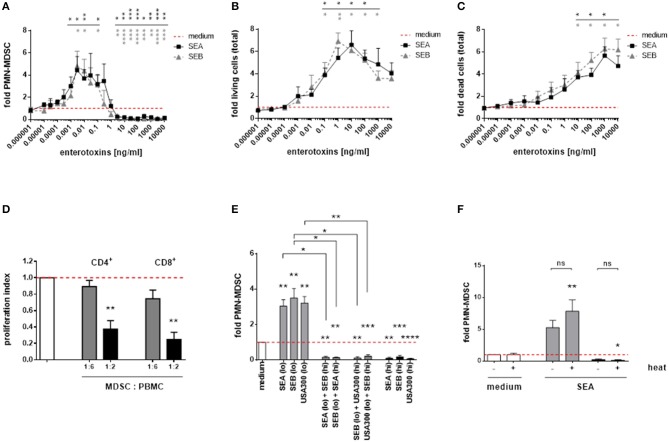
Staphylococcal enterotoxins dose-dependently modulate *PMN-like* MDSC formation, proliferation and cytotoxic effects in PBMC. **(A)** *PMN-like* MDSC were quantified by flow cytometry. **(B)** Proliferation of total PBMC was assessed by trypan blue staining. **(C)** Cytotoxic effects in total PBMC cultures was measured by PI staining and subsequent flow cytometry. **(D)** T-cell suppression. *PMN-like* MDSC were induced by stimulation of PBMC with SEA (0.1 ng/ml) for 7 days, and the CFSE proliferation assay was run as described in Figure 1. Bars represent the proliferation index. **(E)** Cross-inhibition of MDSC formation by enterotoxins. High enterotoxin concentrations inhibited *PMN-like* MDSC induction mediated by *S. aureus* supernatants or by other enterotoxins. PBMC were stimulated with high concentrations (3% of USA300 or Newman supernatants, 5 ng/ml of SEA, or SEB, shown as black bars) and low concentrations (0.01% of USA300 or Newman supernatants, 0.1 ng/ml of SEA or SEB, light gray bars), respectively. Mixed stimulations are shown as dark gray bars. **(F)** Heat-stable nature of SEA. Both SEA-mediated induction and suppression of *PMN-like* MDSC was not affected by heat-treatment. SEA was heated at 80°C for 20 min prior to stimulation at 0.1 ng/ml (gray bars) or at 10 ng/ml (black bars). Data are presented as means ± SEM. Differences between stimulated samples and medium controls were analyzed by a one-sample *t*-test. Differences between single and mixed stimulations **(E)** or between heat-treated and non-treated samples **(F)** were analyzed by a Mann-Whitney test. Statistical results are shown in colors referring to the respective curves. *p < 0.05; **p < 0.01; ***p < 0.001; ****p < 0.0001; ns–not significant.

Consistent with our results obtained for complete *S. aureus* supernatants, staphylococcal enterotoxin A (SEA), and enterotoxin B (SEB) dose-dependently modulated *PMN-like* MDSC. The MDSC modulating potencies of SEA and SEB were similar (Figure 5A). Furthermore, both enterotoxins exerted profound cytotoxic and proliferative effects on PBMC, with *PMN-like* MDSC numbers declining and total PBMC numbers increasing at higher concentrations. The patterns of dose-dependent changes of MDSC and total PBMC numbers mediated by enterotoxins resembled those obtained from stimulations using complete *S. aureus* supernatants (Figures 5B,C). Low SEA concentrations (0.1 ng/ml) induced functional, T-cell suppressive PMN-MDSC, as shown by CFSE proliferation assays (Figure 5D). In addition, when simultaneously applied, MDSC inducing concentrations were outweighed by suppressive concentrations of *S. aureus* supernatants and staphylococcal enterotoxins, respectively (Figure 5E). The MDSC-modulating effects of enterotoxins were heat-stable, as shown for SEA (Figure 5F).

### Mutation of enterotoxin genes diminish MDSC modulating capacities of *S. aureus* supernatants

**Figure 6:**
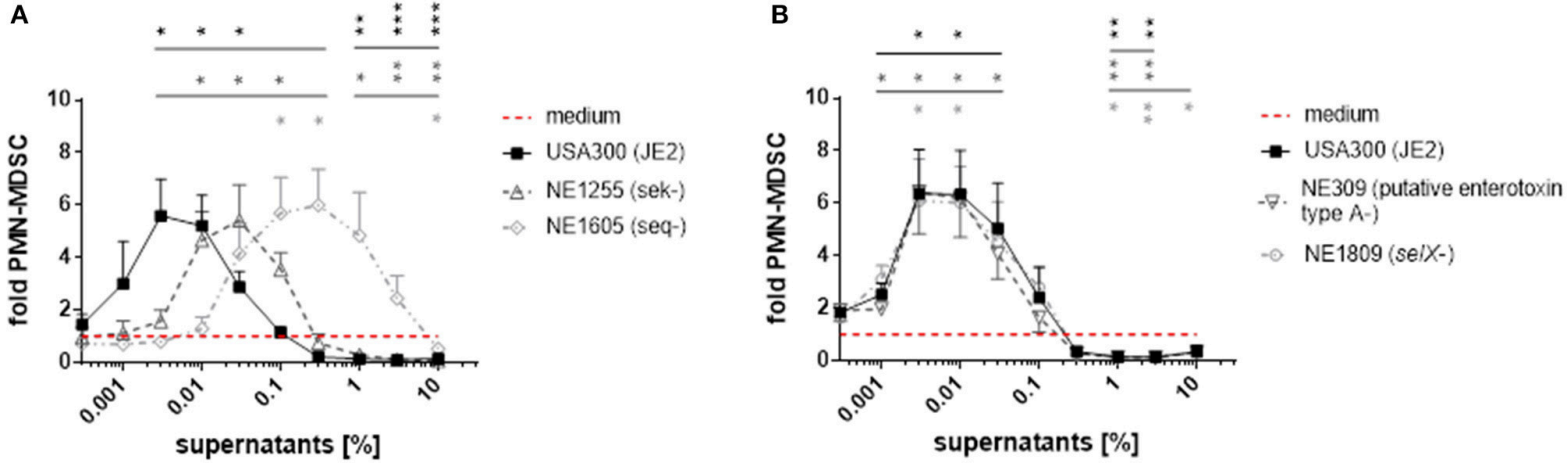
Mutations in *sek* and *seq* affect *PMN-like* MDSC modulating activities of *S. aureus* supernatants. For stimulation of PBMC, supernatants from JE2 strains harboring transposon mutations in the genes *sek, seq, selX* and in a putative “type A”enterotoxin gene were used. **(A)** *PMN-like* MDSC inducing and inhibiting capacities of JE2 supernatants were affected by mutations in the *sek* and *seq* genes. **(B)** Mutations in *selX* and in the gene encoding the “type A”enterotoxin had no effect on *PMN-like* MDSC levels. Data represent means ± SEM. *p < 0.05; **p < 0.01; ***p < 0.001.

To further elucidate the role of enterotoxins in formation of *PMN-like* MDSC, we used transposon mutants derived from the *S. aureus* strain JE2, a derivative of USA300 LAC that had been established by plasmid curing. Strain JE2 and its mutants were taken from the Nebraska transposon mutant library of *S. aureus* strains, which encompasses around 2,000 mutants, each harboring a transposon insertion in a distinct single gene (Fey et al., 2013). The JE2 genome includes the *sek* and *seq* genes and a *selX*-homolog gene, encoding staphylococcal enterotoxin K (SEK), staphylococcal enterotoxin Q (SEQ), and the enterotoxin-like protein SElX, respectively. It also contains a gene encoding a protein with homology to other enterotoxins, referred to as ‘putative enterotoxin ‘type A’. When used for stimulation of PBMC, the inactivated *seq* gene resulted in a 100-fold lower potency of *S. aureus* supernatants to modulate MDSC, whereas inactivated *sek* led to an ~10-fold lower potency (Figure 6A). In contrast, transposon mutagenesis of genes encoding SElX or the putative “type A” enterotoxin, which is not a homolog of SEA as determined by blastp analyses, did not affect MDSC levels compared to wild-type (Figure 6B).

Our results suggest that staphylococcal enterotoxins are crucial components in *S. aureus* supernatants for the modulation of MDSC levels, with staphylococcal enterotoxin Q being the strongest MDSC modulating enterotoxin in USA300 supernatants.

### Modulation of *PMN-like* MDSC correlates with enterotoxin expression

We examined the enterotoxin contents semi-quantitatively in the supernatants of USA300, Newman, COL, and NCTC 8325 by using mass spectrometry. After trypsin digestion of the proteins, the resulting peptides were aligned with protein sequences derived from all *S. aureus* strains available in UniProt database. In Table 2, all proteins detected in the supernatants and identified as staphylococcal enterotoxins (SEs) or enterotoxin-like proteins (SEls) are listed. Peptides derived from eight SEs and SEls could be identified in the culture supernatants of strain COL, where SEB turned out to be the dominant enterotoxin. In Newman, peptides derived from four SEs or SEls could be detected, with SEA and SelX being the most prominent enterotoxins. USA300 was found to express only three types of SEs or SEls, respectively. SEQ appeared to be the major enterotoxin in USA300, followed by SEK, in line with the substantially reduced PMN-MDSC-inducing capacity of the *seq-* mutant of USA300 substrain JE2 (Figure 6A). Only low amounts of enterotoxin-derived peptides were obtained from NCTC 8325 supernatants.

**Table 2:** Quantification of enterotoxins and enterotoxin-like proteins secreted by *S. aureus* strains.

					**LPQ intensities**			
**Locus tag**	**Gene**	**Protein**	**aa**	**kDa**	**USA300**	**NCTC 8325**	**COL**	**Newman**
NWMN_0400	*sea (entA)*	SEA	257	29.7	0	0	0	1,259,600,000
SACOL0907	*seb (entB)*	SEB	266	31.4	0	0	49,282,000,000	0
G8RCC1 *[b]*	*[a]*	SEB-like *[a,d]*	*[a]*	*[a]*	0	1,328,100	0	7,862,500
SAUSA300_0800, SACOL0886	*sek*	SEK	242	27.8	118,420,000	0	4,856,160,000	7,460,700
Q6G7U0 *[b]*	*sek2*	SEK2 *[e]*	242	27.8	0	0	7,080,700	0
SAUSA300_0801, SACOL0887	*seq, sei*	SEQ, SEI *[f]*	242	28.2	849,400,000	0	2,840,600,000	0
SAZ172_0832 *[c]*	*sel*	SEL	256	29.8	0	0	741,110,000	0
HMPREF0769_11854 *[c]*	*seu*	SEU	261	30.5	0	0	2,741,200,000	0
CH52_09990 *[c]*	*yent1*	Yent1 *[g]*	131	15.3	0	0	42,015,000	0
SAUSA300_0370, SACOL0442, NWMN_0362	*selX*	SElX	203	23.2	16,568,000	0	26,516,000	3,726,600,000
Total LFQ					984,388,000	1,328,100	60,536,681,700	5,001,523,200

*Supernatants derived from TSB overnight cultures were analyzed for their relative amounts of enterotoxins and enterotoxin-like proteins by mass spectrometry. The secreted proteins were proteolytically digested and the resulting peptides were aligned with protein sequence entries in the UniProt databases. Shown are all proteins identified as enterotoxins in UniProt databases by means of peptide alignments. Locus tags, designation of genes and proteins, sizes and molecular weights (both of which including signal sequences) refer to the proteins detected in the supernatants of the respective strains and are taken from UniProt databases and from genomic S. aureus databases (GenBank). If not available, locus IDs and designations were deduced from homologous proteins of other strains, or were listed as UniProt ID. High LFQ intensities correspond with high expression levels. LFQ, label free quantification. [a] Data not available in UniProt or in GenBank databases. [b] UniProt ID (locus tag not available in S. aureus databases). [c] Locus entries taken from homologs of strains Z172 (SAZ172_0832), MN8 (HMPREF0769_11854) and 502A (CH52_09990). [d] The obtained peptide sequences are linked to UniProt entry G8RCC1 which is the SEB protein. Since the seb gene is absent from the annotated NCTC 8325 and Newman genomes, the putative protein is termed “SEB-like” in this study. [e] The peptide sequences refer to protein SEK2, sharing 99% homology and 97% identity with SEK from COL using blastp analyses. In several strains, SEK2 is also designated SEK. [f] The proteins SEQ in USA300 and SEI in COL share 98% homology and 97% identity as determined by blastp analyses. [g] Yent1 is a homolog fragment of SEU in several S. aureus strains*.

Our results show that *S. aureus* strains with the highest functional capacity of MDSC modulation displayed the highest total enterotoxin content determined by mass spectrometry. An overall LFQ intensity of approximately 60.5 billion units was determined for enterotoxins in COL supernatants which induced maximum *PMN-like* MDSC levels at concentrations of 0.001% (see Figure 2), followed by Newman (~5.0 billion units; maximum induction at 0.003%), USA300 (~1.0 billion units; maximum at 0.01%), and NCTC 8325 (~0.01 billion units; 10% of supernatant necessary for maximum MDSC induction). It has to be taken into account that for mass spectrometry analysis it was necessary to switch from RPMI to TSB medium for staphylococcal cultures in order to yield high enough bacterial density for protein analyses. Therefore the enterotoxin contents are not directly correlated between these two growth conditions as the growth rate of *S. aureus* influences virulence factor/enterotoxin production (Derzelle et al., 2009).

### Staphylococcal enterotoxins substantially interfere with MDSC-inducing signals

**Figure 7:**
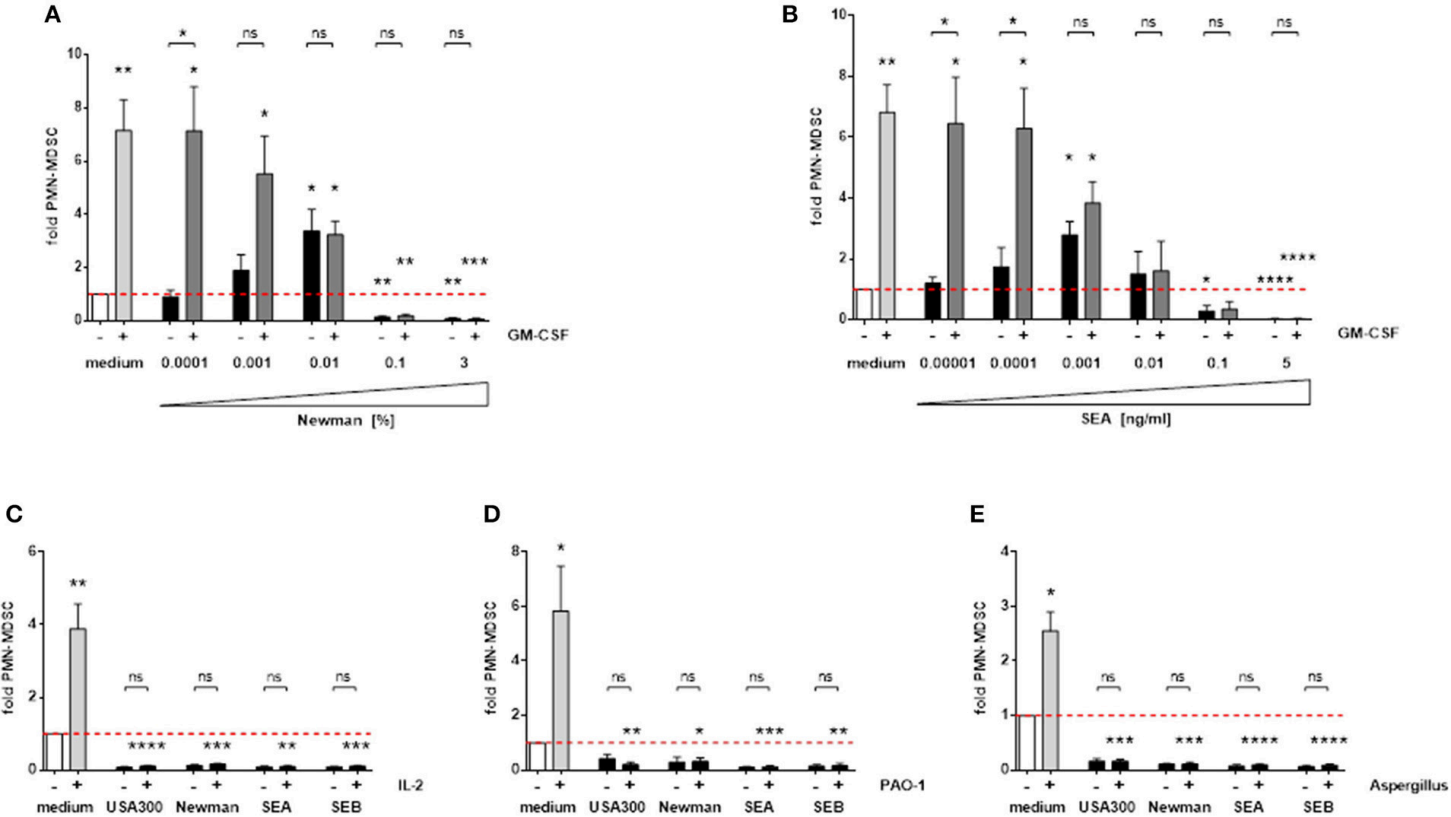
*S. aureus* supernatants and enterotoxins interfere with other MDSC-inducing substances. PBMC were stimulated simultaneously with 10 ng/ml GM-CSF and increasing concentrations of Newman supernatants **(A)** or SEA **(B)**. High concentrations of *S. aureus* supernatants (3%) or enterotoxins (5 ng/ml) were used for co-stimulation with IL-2 (100 U/ml) **(C)**, *P. aeruginosa* PAO-1 supernatants (1%) **(D)** or *Aspergillus fumigatus* lysates (10 μg/ml) **(E)**. Data represent means ± SEM. Differences between stimulated PBMC and medium controls (white bars) were analyzed by a one-sample *t*-test. Differences between single and mixed stimulations were analyzed by a Mann-Whitney test *p < 0.05; **p < 0.01; ***p < 0.001; ****p < 0.0001; ns–not significant.

MDSC are induced by various endogenous growth factors, cytokines, and microbial stimulants. GM-CSF is an important MDSC inducing growth factor, which led to an approximately 7-fold increase in PMN-MDSC numbers in our experiments. The strong MDSC-inducing capacity of GM-CSF is dose-dependently suppressed by *S. aureus-*derived supernatants and enterotoxins (Figures 7A,B). In addition, PMN-MDSC suppressing concentrations of *S. aureus* supernatants and enterotoxins also abolished the substantial MDSC-inducing capacities of IL-2 and of preparations derived from several microbes, e.g., *Pseudomonas aeruginosa* supernatants or *Aspergillus fumigatus* lysates (Figures 7C–E). Hence we propose that *S. aureus* enterotoxins have a dominant effect on a diverse range of MDSC-inducing factors.

## Discussion

In this study we systematically analyzed the generation of MDSC upon stimulation with multiple *S. aureus* strains and their major virulence factors. We demonstrate for the first time that staphylococcal enterotoxins dose-dependently modulate the generation of *PMN-like* MDSC. Lower concentrations of enterotoxins induced *PMN-like* MDSC, whilst increased concentrations inhibited the generation of *PMN-like* MDSC and strikingly abolished MDSC induction by GM-CSF, *P. aeruginosa, A. fumigatus* and IL-2.

These concentration-dependent results of staphylococcal enterotoxins are exceptional when compared to other pathogen-MDSC interactions. So far, all reported pathogens and associated virulence factors either led to an induction or inhibition of MDSC (Ost et al., 2016). Examples for induction include, but are not limited to, *S. aureus, Pseudomonas aeruginosa*/flagellin, *Klebsiella pneumonia*, or pathogenic fungi (Poe et al., 2013; Rieber et al., 2013, 2015; Heim et al., 2014; Skabytska et al., 2014; Tebartz et al., 2015; Szabo et al., 2016). An inhibitory effect as seen with staphylococcal enterotoxins at increased concentrations has been observed for the TLR3 agonist Poly (I:C) (Ho et al., 2015). Stimulation of TLR9 with CpG oligonucleotides induced maturation of M-MDSCs and led to a loss of their immunosuppressive function (Zoglmeier et al., 2011; Shirota et al., 2012). A combination of TLR7, 8 and 9 ligands enhanced anti-tumor responses by NK cells and cytotoxic T-cells and reduced MDSC frequency (Zhao et al., 2014).

How do staphylococcal enterotoxins interact with MDSC at the molecular level? Is there a MDSC-specific cytotoxic mechanism? It has been demonstrated that staphylococcal enterotoxins primarily interact with professional antigen-presenting cells via MHC class II leading to their activation and production of proinflammatory cytokines and chemokines (Pinchuk et al., 2010). To our knowledge, no direct cytotoxic mechanism of staphylococcal enterotoxins has yet been identified. However, MDSC might exhibit unique receptors or an otherwise specified susceptibility to cytotoxic staphylococcal enterotoxins compared to other myeloid cells and lymphocytes. Another possible mode of action of these super-antigens would be indirect, e.g., by massive cytokine secretion of the cell environment surrounding MDSC, a scenario sometimes called cytokine storm, which could lead to accelerated apoptosis/necroptosis of MDSC. With rising toxic effects, *PMN-like* MDSC declined whereas total PBMC numbers initially further increased. However, due to the gating region used in our experiments for assessment of *PMN-like* MDSC numbers we deem it unlikely that the decrease of MDSC is merely caused by concomitant increase of other cell types and may therefore only be a relative decrease. The only previous report on the interaction of staphylococcal enterotoxins and MDSC by Szabo et al. described a swift influx of PMN-MDSC in the mouse liver after inoculation of staphylococcal enterotoxin B. The authors concluded that this rapid influx was not due to proliferation or generation of MDSC but rather due to homing signals to the liver from the bone marrow. The exact pathways, however, are a matter of future investigation (Szabo et al., 2016). A cytotoxic effect of enterotoxins on MDSC was not described in that study.

Superantigens from *S. aureus* trigger exhaustive polyclonal T cell proliferation in the infected organism. Our results point to a reinforcing pathway that supports this mechanism by abrogating T-cell suppressive MDSC if the bacterial cell density is high enough and enterotoxins are secreted in considerable concentrations. This mechanism might be reminiscent of the well-defined quorum sensing system of *S. aureus*, that is cell density-dependent gene regulation mediated by the accessory gene regulator (Agr) system (Peschel and Otto, 2013). In our study we did not dissect the signaling pathways that are involved in the interaction between enterotoxins and MDSC. However, this was out of the scope of this initial study but is under current investigation within our group. In addition, the evaluation of our findings in *in vivo* models and differentiation of the interaction between *S. aureus* and MDSC between different organ compartments will be of special importance for future studies.

Activated CD4 T cells have been shown to promote the pathogenicity of *S. aureus* (Parker et al., 2015). Therefore, we propose that the inhibition of MDSC generation represents a newly defined pathogenic mechanism of *S. aureus*. Blocking enterotoxins in invasive *S. aureus* infections or adoptive transfer of MDSC could ameliorate hyperinflammatory reactions to *S. aureus*. On the other hand, enterotoxins could be of interest for therapeutic purposes in preventing the formation of MDSC. Especially in tumor patients, where MDSC are consistently induced and weaken the innate and adaptive anti-tumor immune response, inhibition of MDSC would be advantageous. Fine-tuning the biphasic relation between *S. aureus* enterotoxins and MDSC *in vivo* will be challenging and will require exhaustive studies in animal models to prevent harmful effects. A rather dominant effect of enterotoxins on MDSC-inducing signals has been shown in our co-culture experiments with tumor-associated GM-CSF (Lechner et al., 2010), IL-2 (Rodriguez et al., 2009), and Pseudomonas- (Rieber et al., 2013) and Aspergillus- (Rieber et al., 2015) derived MDSC-inducing molecules. However, for therapeutic approaches it would be crucial to better define the responsible domain of enterotoxins for this MDSC abrogating effect first and biochemically engineer these molecules to clear off existing severe side effects of these toxins. The interaction between *S. aureus* enterotoxin related abrogation of MDSC and Pseudomonas related induction of MDSC (Rieber et al., 2013) again highlights the specific pathophysiological conditions for the frequent co-infections with different pathogens in multifaceted diseases like cystic fibrosis.

Taken together, we identified staphylococcal enterotoxins as main modulators of MDSC generation. The interaction of staphylococcal enterotoxins with myeloid-derived suppressor cells might play an important role in the overshooting inflammatory reaction frequently seen in systemic *S. aureus* infections and might represent a novel therapeutic target in *S. aureus* infections and beyond.

## Ethics statement

This study was carried out in accordance with the recommendations of the ethics committee of the University of Tuebingen. All subjects gave written informed consent in accordance with the Declaration of Helsinki and the ethics committee of the University of Tuebingen approved the study protocol.

## Author contributions

HS designed and performed experiments, analyzed the data, and co-wrote the manuscript. MO and RM performed experiments and analyzed the data. AS performed and supervised experiments, discussed the data, and revised the manuscript. DN discussed the data and helped with experiments. IS helped with experiments. AV and BM performed protein analysis. DK and CW generated specific culture supernatants and purified *S. aureus* products and discussed the data. AH, RH, FG, and AP provided guidance in the study and revised the manuscript. DH co-designed the study, discussed the data, and revised the manuscript. NR co-designed the study, supervised the experiments, analyzed, and discussed the data and co-wrote the manuscript.

### Conflict of interest statement

The authors declare that the research was conducted in the absence of any commercial or financial relationships that could be construed as a potential conflict of interest. The reviewer JM and handling Editor declared their shared affiliation.
