# Supplementary figures and images for: Staphylococcal Enterotoxins Dose-Dependently Modulate the Generation of Myeloid-Derived Suppressor Cells
Source: Front Cell Infect Microbiol. 2018 Sep 13;8:321. doi: 10.3389/fcimb.2018.00321 (PMC6146041; doi:10.3389/fcimb.2018.00321)

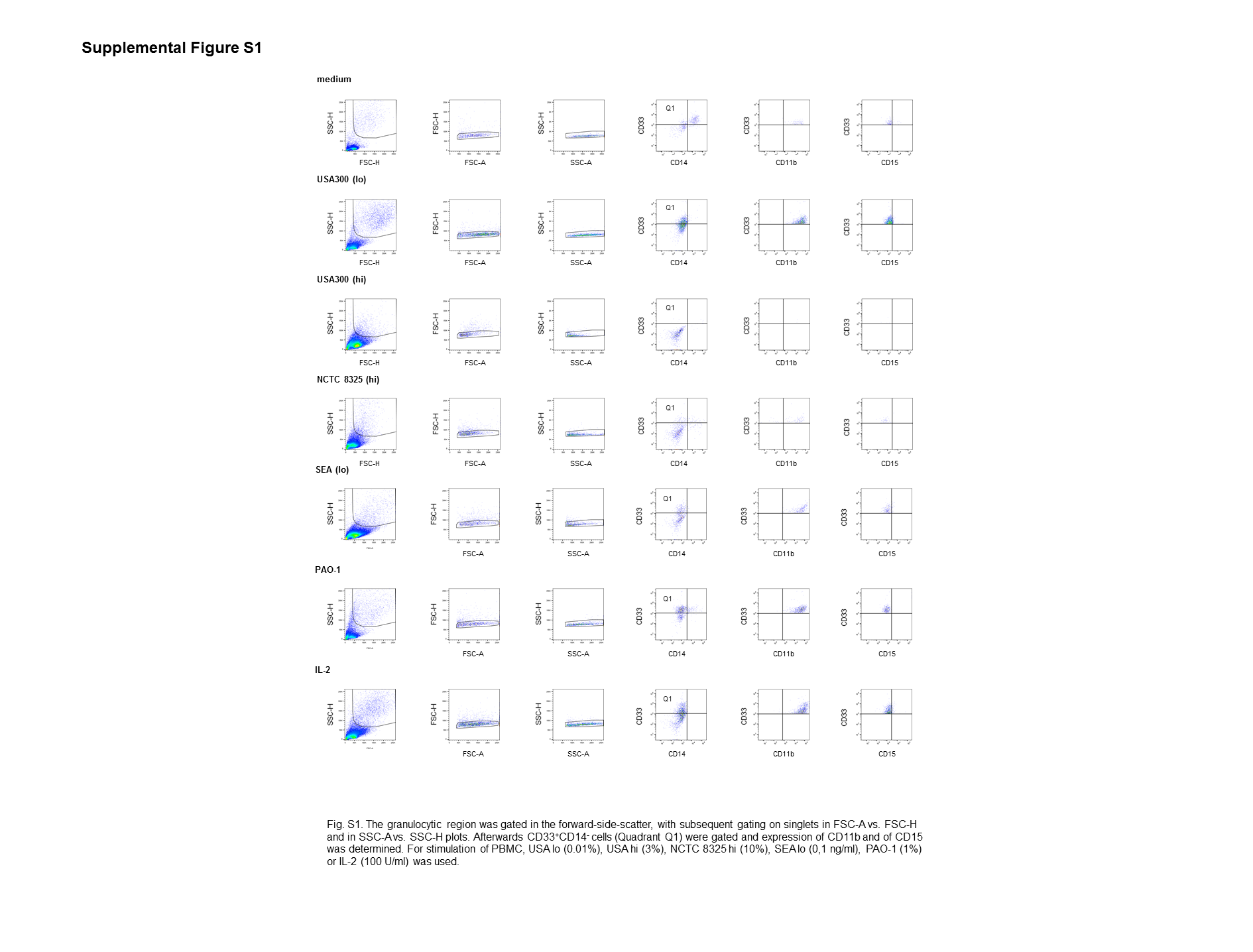

Supplement: Supplementary file 1 [file Image_1.TIF]

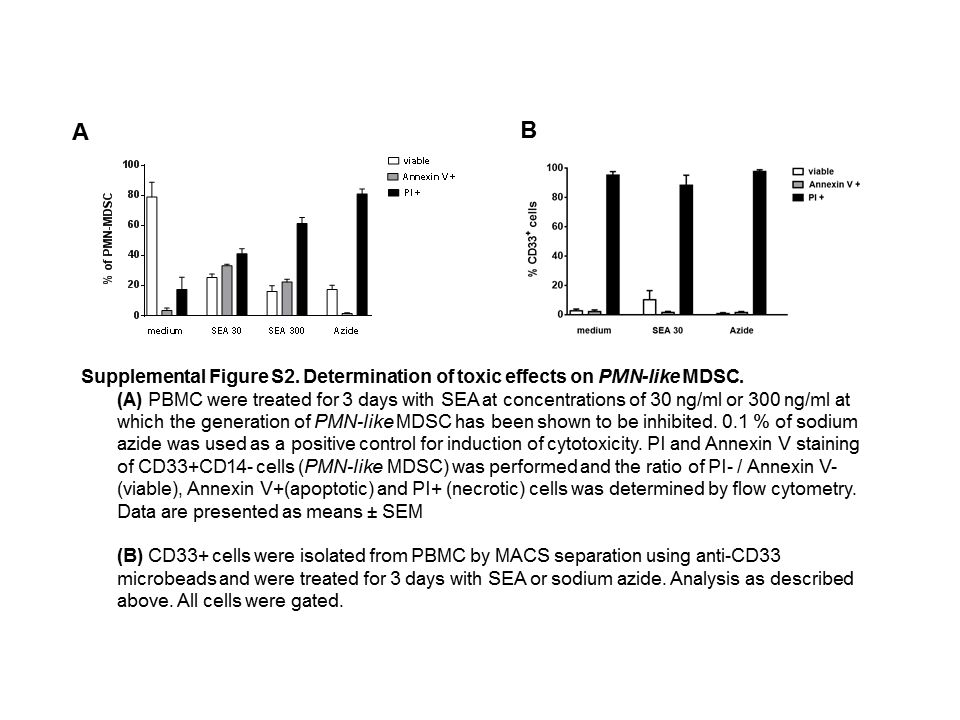

Supplement: Supplementary file 2 [file Image_2.TIF]
